# Identifying the Weaker Function Links in the Hazardous Chemicals Road Transportation System in China

**DOI:** 10.3390/ijerph18137039

**Published:** 2021-07-01

**Authors:** Laihao Ma, Xiaoxue Ma, Jingwen Zhang, Qing Yang, Kai Wei

**Affiliations:** 1 Marine Engineering College, Dalian Maritime University, Dalian 116026, China; malaihao@dlmu.edu.cn; 2 Public Administration and Humanities College, Dalian Maritime University, Dalian 116026, China; zhangjingwen2021@126.com (J.Z.); yangqing@dlmu.edu.cn (Q.Y.); weikai@dlmu.edu.cn (K.W.)

**Keywords:** hazardous chemicals, hazardous chemicals road transportation system, risk analysis, FRAM

## Abstract

Safety of the hazardous chemicals road transportation system (HCRTS) is an important, complex, social, and environmental sensitive problem. The complexity, dynamics, and multi-link features of HCRTS have made it necessary to think beyond traditional risk analysis methods. Based on the relevant literature, Functional Resonance Analysis Method (FRAM) is a relatively new systemic method for modeling and analyzing complex socio-technical systems. In this study, a methodology that integrates FRAM, fuzzy sets, and risk matrix is presented to quantitatively assess the risks factors representing failure function links in HCRTS. As the strength of function links can be illustrated by the RI (risk index) of risk factors identified in failure function links, 32 risk factors representing 12 failure function links were first identified by accident causes analysis and the framework of FRAM. Fuzzy sets were then utilized to calculate the weight of the likelihood and consequence of the risk factors. Finally, according to the assessment results of the identified risk factors by a two-dimensional risk matrix, the weaker function links in the whole HCRTS chain were identified. HCs road companies, regulatory authorities, relevant practitioners, and other stakeholders should pay more attention to these links.

## 1. Introduction

Hazardous chemicals (HCs) are widely used in the production activities of chemical industries and many other manufacturing industries, which not only lay an economic foundation, but are also an indispensable part of production and life [1,2,3]. China is the world’s largest producer of HCs, accounting for about 40% of the world’s chemical production capacity [4]. Most of the chemical industry clusters of basic raw materials are located in western China, and most of the sale places and downstream deep-processing enterprises are concentrated in the eastern coastal areas. As a result, HCs industries in China have obvious characteristics of the separation of production and marketing, which determines that more than 95% of HCs need to be transported [5]. As one of the most important HCs transport modes in China, road transportation moves more than 80% of the total HCs volume each year; nearly 3 million tons of HCs are transported by road every day, which is far higher than the proportion (approximately 60%) of HCs transported by road in Europe [6]. By the end of 2019, there were about 130,000 HCs transport enterprises in China, with 1.66 million employees, including drivers, escorts, and stevedors, and 380,000 HCs transport vehicles [7]. Compared to general cargos, the HCs have some unique physical and chemical properties (explosive, corrosive, toxic, radioactive, etc.). The transportation of HCs is also known as a “mobile bomb”—this is because the accidents caused by HCs during road transportation may result in catastrophic loss of lives and money, as well as damage to the environment. According to the China Chemical Safety Association, the number of HCs accidents decreased significantly after the explosion in Tianjin port in 2015, but there were 5513 HCs accidents in China from 2013 to 2019; unfortunately, the trend of HCs accidents has begun to increase in recent years [8]. The increasing volume and type of HCs threatens road traffic safety and poses great risks to the public, indicating that the risk prevention and control of HCRTS is still facing severe challenges.

Risk analysis is considered to be an effective way of devising mitigation measures to avoid accidents, which have greatly concerned the public, HCs transportation enterprises, and corresponding government management departments [9]. The Ministry of Transport of the People’s Republic of China (MTPRC), Ministry of Emergency Management of the People’s Republic of China (MEMPRC), State Administration for Market Regulation (SAMR), Ministry of Industry and Information Technology of the People’s Republic of China (MIITPRC), Ministry of Ecological and Environment of the people’s Republic of China (MEEPRC), and the Ministry of Public Security of the People’s Republic of China (MPSPRC) have issued a series of management documents and have deployed special inspection activities to improve the safety performance of HCRTS, but the effects of these risk prevention and control measures have not been satisfied. There are multiple functional links in HCRTS, involving different government management departments [10]. Some risk management efforts conducted by these departments only focus on a certain segment or a functional link and there is no effective collaborative regulatory force for the whole of HCRTS, which may lead to regulatory gaps and breakpoints [11]. More importantly, the occurrence of major accidents has highlighted the urgent need for advancement of methods or tools for risk evaluation and safety management in HCRTS, since conventional ways of safety management focusing on failures and treating them as a cause-and-effect relationship [12,13], are less effective in controlling the risks with uncertainty, intractability, and complexity in HCRTS. In HCRTS, hazards and risks have evolved due to linear risk propagation paths not being followed, thus resulting in emergent accidents. This requires a more accurate and systematic method to be developed to manage HCRTS risks effectively. FRAM is a systemic accident analysis method based on resilience engineering that has been proven to be an effective tool [14,15,16]; it can provide a better understanding and management of complicated systems, where processes are non-linear and outcomes are emergent, like HCRTS. In HCRTS, the failure of links between different functions could lead to undesired or unexpected consequences. FRAM describes the dynamic process of a system with different functions, which is preferred for the analysis of HCRTS. To the best of our best knowledge, there has been little research on the development of a functional network model of HCRTS, and to identify and analyze the safety risks in different functional links of HCRTS under multiple regulatory authorities. However, FRAM is designed as a framework and often used as a qualitative method, which is incapable to quantify the risk and failure function links.

To fill this gap, in the paper, a methodology integrating FRAM, fuzzy sets, and risk matrix was proposed to assess the risk factors representing corresponding failure function links in HCRTS. This study is formulated as follows: Section 2 is devoted to the literature review of the methods of accident-causing models. Section 3 includes an introduction to HCRTS management in China. A detailed description of the proposed method is presented in Section 4. Section 5 is devoted to application of the proposed method in HCRTS. Section 6 provides the conclusions and outline of future research tasks.

## 2. Literature Review

Transportation safety of HCs has been a long-term concern and continuous research topic for many scholars because of its high social concern [17,18]. Accident analysis is an important tool that can be useful for improving and/or optimizing the safety level of HCRTS [19]. In the past five decades, a large number of studies have been conducted on accident analysis. With the development of socio-technical systems, cognition of the nature of accidents has gradually deepened, and the accident-causing theory has been rapidly developed [20,21]. According to the literature, mainstream accident analysis methods can be categorised as simple linear accident-causing model, complex linear accident-causing model, and dynamic system accident-causing model. This section is devoted to the literature review on these approaches, and presentation of their advantages and disadvantages.

### 2.1. Simple Linear Accident-Causing Model (SLAM)

The application of the SLAM is to evaluate individual factors in a linear relationship [22]. In the SLAM, it is generally believed that a possible reason behind the undesired or unexpected safety results is system components failures, errors, or faults. Therefore, this way of thinking focuses on the components in the system and their failures. As a representative, in the causal chain theory of accidents [23], the Domino model was developed to describe various factors that caused accidents, considered that an accident is not caused by an isolated event, but is the result of a series of discrete events. The genetic and social environment, human factors, unsafe behavior and state, accidents, and injuries constitute the five factors of Domino model. The occurrence of one factor will trigger a chain reaction, and subsequent domino factors will occur one after the other. When the last domino falls, it will cause damage. On this basis, linear models such as failure mode and effect analysis (FMEA) [24], Fault Tree Analysis (FTA) [25,26], and Event Tree Analysis (ETA) [27,28] were developed. However, these models are effective in analyzing the simple accidents caused by a single factor, but it is not suitable to explain accidents involving the interaction of multiple risk factors in complex systems, like HCRTS. 

### 2.2. Complex Linear Accident-Causing Model (CLAM)

With deepening cognition of the accident-causing model, it is generally acknowledged that simple linear thinking has defects [29,30]. Even in a relatively well-managed work environment, serious events may occur, and these events usually involve multiple serial or parallel event sequences. CLAM aims to explain the interaction of multiple risk factors and the causality of complex system accidents. As a representative, the Swiss Cheese Model [31] was established based on epidemiological theory, considered that the accidents are caused by the joint action of the loopholes in the organization, operation, and other layers. Each layer is represented by a piece of cheese, and the holes on the cheese represent the loopholes on each layer. When all the holes on the cheese are arranged in a straight line, it means that all the defense measures have failed, which will lead to dangers or accidents [32]. From the Swiss cheese model, it can be concluded that all kinds of loopholes lead to accidents, including unsafe behaviors and potential factors, which make up for the defects of the linear model in describing the environment of the system. The Swiss Cheese Model promotes studies on human factors in the accident analysis; based on this inspiration, the Human Factor Analysis and Classification System (HFACS) model was proposed [33], which further divides the hierarchy of human factors into four levels, including unsafe behavior, precondition of unsafe behavior, unsafe supervision, and organizational management. Each level corresponds to one layer of the Swiss Cheese Model. The advantage of HFACS is that it considers the organizational factors in the cause of accidents, which makes it an effective tool for the analysis of human factors in aviation accidents [34], and has been widely used in maritime [35], HCs transportation [36], mining [37], and other fields. However, the two approaches still consider that accidents are a result of a series of mutual causal events, although they do consider the complexity of the accident process and could reveal the relationship between potential cause and direct cause in the accidents. The interactions between various factors in the complex system are still not well-explained because of the ambiguity in the definition of loopholes and each level/layer.

### 2.3. Dynamic System Accident-Causing Model (DSAM)

In a complex socio-technical system, risk factors have complex links, non-linear interactions, and random or uncertain effects; the linear causal model mentioned above will not be able to effectively deal with complex systems with interactive characteristics [38]. Hence, the DSAM is devoted to describing the accident process as a complex and multi-interactive event network based on system theory. Unlike the SLAM and CLAM, according to the DSAM, accidents are not caused by a single component failure or an operator’s error [39], but by the complex interaction of operator, technology, organization, management, environment, and other factors [40]. In other words, DSAM considers the functions and interactions of the whole system, as well as the performance variability or control levels of the system, instead of analyzing single components of the system. AcciMap, STAMP, and FRAM are the representatives of DSAM, and widely applied in the many fields.

AcciMap integrates the cause-and-risk management framework, which is used to analyze a series of interactive events and decision-making processes that occur in the whole socio-technical system [41], focusing on describing the potential contributory failure factors in the system from six organizational levels, including the government, regulators, company, company management, staff, and workflow. Though AcciMap can explain the causes of accidents systematically, this method is still affected by sequential event chains [42].

The STAMP model, based on the system theory and control theory, considers that the accident occurs because the interaction between system components violates certain constraints [42]. In other words, the risks in the system are regarded as emergences, which come from the interaction between system components. Safety problems could be transformed into a control problem from this point of view, and the priority of system safety is no longer to prevent component failure, but to impose safety constraints on control behavior. Although the establishment and analysis of the STAMP model is from the perspective of the system, there is insufficient efficiency in the dynamic analysis of complex socio-technical system safety [43].

FRAM is another systemic accident analysis method developed from the fundamentals of resilience engineering for the risk identification and accident analysis of complex socio-technical systems [14]. Unlike most traditional risk assessment methods that focus on the root cause of failures and decompose a system into components, FRAM provides a framework that describes the dynamic process of a system with different functions. Compared to other methods, FRAM assumes that successes and failures happen for the same reason, focusing on understanding how functions are coupled and how the variability of daily operations/activities can lead to undesired and unexpected results. As a new approach for safety and risk management over the past decades, FRAM has proven its efficiencies and values in aviation safety [44], healthcare [45], maritime [46], construction [47], railway [48], etc.

To sum up, fundamentally speaking, SLAM, DLAM, AcciMap, and STAMP models are based on the perspective of causality to explain the cause of accidents, which has certain limitations in the analysis of complex coupling accidents. Different from the previous system model, which focuses on system failure, FRAM focuses on how the system functions can be implemented correctly from the perspective of normal operation. In the present study, FRAM was utilized to construct the operation network of HCRTS, by which the failure function links can be extracted efficiently with the help of identified risk factors.

## 3. Background: Management of HCRTS in China

**Figure 1:**
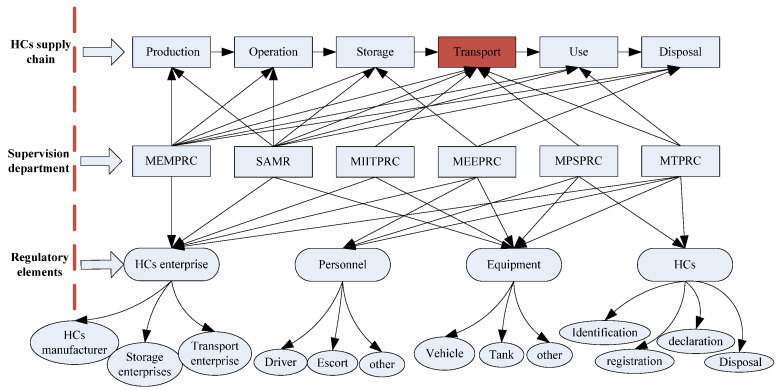
Safety supervision network of HCs.

Law and regulation systems related to HCRTS in China include three levels [49]. The first is the Production Safety Law of the People’s Republic of China (PSLPRC), which is the most fundamental law in the field of safety production, and also the most basic legal basis for the supervision of HCRTS. The second is the Regulations on the Safety Management of HCs (RSMHC) formulated and promulgated by the State Council of the People’s Republic of China. It is to be noted that, as the current administrative regulation for HCs, RSMHC not only defines the responsibilities of MTPRC and transportation enterprises in the transportation of HCs, but also includes the management requirements of HCs in production, storage, operation, use, and disposal, involving MEMPRC, SAMR, MIITPRC, MEEPRC, MPSPRC, and other government departments in China. The third level for road transport of HCs is the Safety Management Measures for Road Transport of Dangerous Goods (SMMRTDG), which was formally promulgated by MTPRC, MEMPRC, SAMR, MEEPRC, and MPSPRC on 1 January 2020. SMMRTDG set systematic regulations and requirements for the whole chain management of HCs transportation, as shown in Figure 1. The transportation of HCs is only a link in the supply chain of HCs, which includes production, operation, storage, transportation, use, and disposal. Different links of HCs have different regulatory departments and management elements, which interweave with each other and form a complex supervision safety supervision network.

## 4. Materials and Methods

**Figure 2:**
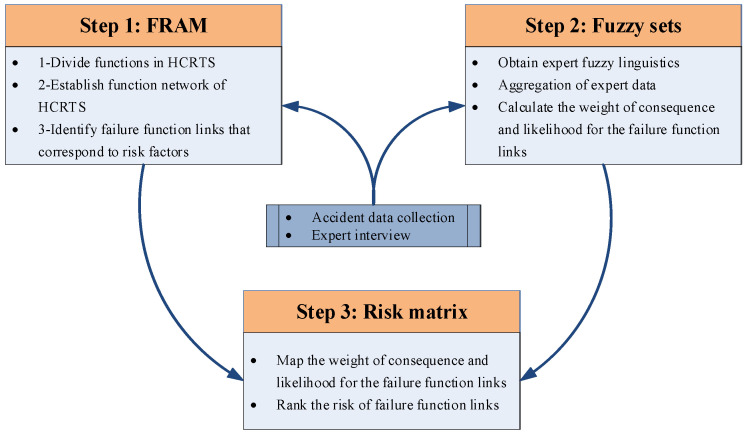
Framework of the proposed methodology.

In this section, a brief background description of the methods used in this study is presented. Figure 2 depicts the framework of the proposed methodology in three steps.

### 4.1. FRAM

**Figure 3:**
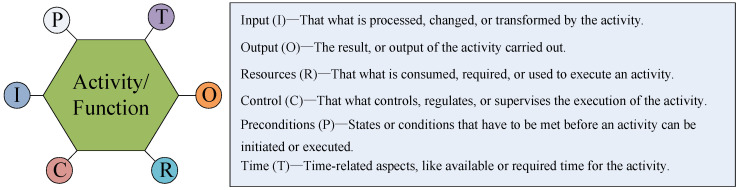
The six parameters of FRAM.

FRAM defines a systemic framework to model complex systems from the perspective of function, and views accidents as an emergent phenomenon of the function’s variability, which plays an increasingly significant role in the development of systemic accident theory. The nonlinear and complex interactions between system functions could be characterized in terms of six parameters displayed in Figure 3—inputs, preconditions, time, resources, controls, and outputs. Input (I) refers to items that are processed, transformed, or needed to start the function. Output (O) is the result of the function, which could be an entity or change of state. Time (T) refers to temporal constraints affecting the function, which is related to start, end, or duration. Control (C) represents the ways that the function is monitored or controlled. Preconditions (P) represent the conditions before the function can be executed. Resources (R) are consumed in the process of generating output. Hence, in the network, the Output upstream function is determined by Precondition, Input, Resource, Control, Time, or Input, and then affects the variability of the downstream function. The coupling can be illustrated graphically by connections of one or more of the parameters from each function.

Function description and nonlinear coupling analysis should be the core and characteristic content of FRAM. Generally, there are four steps to conduct a FRAM analysis [14]:

➢ The first step is to identify and describe the functions required for a successful process of the system, which usually needs to decompose the system process into multiple nodes according to the current operation procedures.
➢ The second step is to identify and characterize the variability of each function from step 1.
➢ The third step is to evaluate how the variability of each function affects the variability of the overall system.
➢ The fourth step is to identify ways to manage performance changes that may not be under control, and to propose methods for managing functional resonance, including not only the measures to reduce the risk, but also the methods to maintain system function; functional resonance effects, especially, should be attenuated by detecting, monitoring, or controlling behaviors.

In the present study, the first step of FRAM was focused upon and utilized to establish the normal operation function network of HCRTS, and to recognize failure function links with the help of risk factors identified by reviewing accident reports.

### 4.2. Fuzzy Risk Matrix

#### 4.2.1. Risk Matrix

The risk matrix is one of the most popular tools in risk assessment. Since risk is usually expressed as a product of the consequence and likelihood of an event under its occurrence, a Risk Index (*RI*) is constructed by the combination of consequence weight and likelihood weight, i.e., *RI* of the *i*th risk factor can be obtained by [50]:

(1) $$RI_{i}=CW_{i}\times LW_{i}$$

In the equation, $CW_{i}$ and $LW_{i}$ represent the consequence weight and likelihood weight of the *i*th risk factor, respectively, which can be calculated by aggregating expert judgement with linguistic expressions under the fuzzy environment in the following section.

#### 4.2.2. Fuzzy Sets

Since experts tend to express their opinions with ambiguous linguistics rather than crisp values, expert judgement can be addressed in linguistic expressions such as low, medium, high, etc. On this basis, a fuzzy number (as membership function) can be adopted to represent the uncertain judgment of an expert and capture parameter vagueness, which is widely adopted in various fields, including accident investigation [51], risk assessment [52,53,54], decision-making [55], etc. The fuzzy numbers signify the degree to which measured elements belong to the preference set, and the linguistic expressions can be transformed in the form of triangular fuzzy numbers (TFN) and trapezoidal fuzzy numbers (TrFNs). TFN is a special type of TrFN with three real figures as its member. According to the definition of a fuzzy number, suppose the fuzzy set is denoted as A=(a,m,n,b), then the member function of TrFN is as follows:

(2) $$\mu_{A}(x)=\left\{\begin{array}{cc} \left(x-a)/(a-m\right) & x\in \left[a,m\right]\\ 1 & x\in \left[m,n\right]\\ \left(b-x)/(b-n\right) & x\in \left[n,b\right]\\ 0 & otherwise \end{array}\right\}$$

In the equation, *a ≤ m ≤ n ≤ b*, *a* and *b* represent the lower and upper bounds of TrFN, respectively. *m* and *n* represent the modal value. If *m = n*, the membership function of TrFN is transformed to TFN, i.e., the form of TFN, B=(a,m,b), can be derived as:

(3) $$\mu_{B}(x)=\left\{\begin{array}{cc} (x-a)/a-m & x\in \left[a,m\right]\\ (b-x)/b-m & x\in \left[m,b\right]\\ 0 & otherwise \end{array}\right\}$$

To convert qualitative linguistic expressions to corresponding fuzzy numbers, Chen and Hwang established a relationship between qualitative terms and their corresponding fuzzy numbers in different scales [56], which enables the transformation of subjective judgements and linguistic expressions into measurable and numerical data. As to the number of verbal expressions, five verb expressions are preferred when an expert makes a suitable judgement [57]. Hence, in the present study, TrFN was employed to calculate the weight of the likelihood and consequence of the risk factors, which were identified from accident data. Then, an expert questionnaire with five-verb expressions ranging from VL, L, M, H, and VH was designed and used to determine the likelihood and consequence of the risk factors in different failure function links of HCMTS. Table 1 provides the linguistic expressions and corresponding TrFNs.

Based on expert judgment with linguistic expressions, the weight of the likelihood and consequence of the risk factors can be obtained by aggregating the TrFNs into crisp values. Suppose that an expert, $E^{k}(k=1,2,3\cdots n)$, states his or her specific viewpoint about the *i*th risk factor by using a predefined set of linguistic expressions, these linguistic expressions can then be transformed into corresponding TrFNs, $P_{i}^{k}=(a_{i}^{k},m_{i}^{k},n_{i}^{k},b_{i}^{k})$, as provided in Table 1. Finally, the defuzzification value $P_{i}$, which in this study was considered as the weight of the likelihood and consequence of the *i*th risk factors, could be procured by Equations (4) and (5):

(4) $$P_{i}^{\prime }=\frac{P_{i}^{1}⊕P_{i}^{2}⊕P_{i}^{3}\cdots P_{i}^{n}}{n}=(a_{i}^{\prime },m_{i}^{\prime },n_{i}^{\prime },b_{i}^{\prime })$$

(5) $$P_{i}=\frac{a_{i}^{\prime }+m_{i}^{\prime }+n_{i}^{\prime }+b_{i}^{\prime }}{4}$$

## 5. Application in HCRTS

This section is devoted to application of the proposed method in the analysis of risk factors, as well as the corresponding failure function links in HCRTS. Firstly, 94 HCs road transportation accident data was collected from MTPRC, MEMPRC, and the National Internet Platform for Public Service of HCs Safety to recognize the risk factors in HCRTS with the guidance of accident investigators, as well as people associated with the accidents. Based on the identification of functions in HCTRS, a normal operation function network was then built by FRAM. Secondly, the corresponding failure function links were identified by the risk factors, which together constitute a two-layer evaluation index system. That is, the strength of the failure function links can be reflected in the evaluation of corresponding risk factors in the link. In the end, a quantitative analysis was performed by the fuzzy set theory and risk matrix.

### 5.1. Idenetifying Failure Functions Links in HCRTS

#### 5.1.1. Decomposing the Functions of HCRTS

**Figure 4:**
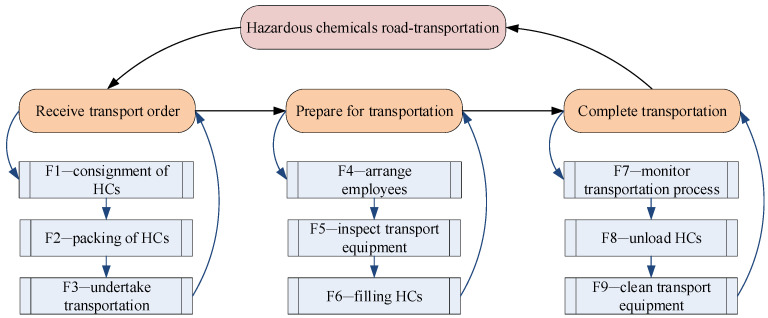
Decomposing the functions of HCRTS by HTA.

Many activities are involved in the road transport of HCs, starting with being delivered by the shipper and ending with being picked up by the consignees. To identify the specific functions which facilitate the establishment of the follow-up network, the hierarchical task analysis (HTA) method, a structured and objective method to describe the hierarchical system of tasks and their subtasks, was employed to decompose the function of HCRTS with the assistance of experts interviewed in HCs road transportation companies. The whole chain of HCRTS covers three processes: receiving the transport order, preparing for transportation, and completing transportation. According to the principle of HTA, each process of HCRTS can be further divided into several specific functions. As a result, a total of nine essential functions were identified in the whole chain of HCTRS by using HTA, as shown in Figure 4; they include F1—consignment of HCs, F2—packing of HCs, F3—undertake transportation, F4—arrange employees, F5—inspect transport equipment, F6—filling HCs, F7—monitor transportation process, F8—unload HCs, and F9—clean transport equipment.

In “F1”, the shipper entrusts a qualified carrier to transport the HCs. They are to be packaged well in “F2” as per the shipper’s requirements. In “F3”, the carrier tells the vehicle fleet and employees to prepare for transportation of the HCs on receiving the transport order. Providing qualified drivers and escorts, and checking whether the transport vehicles and tanks are suitable for HCs transportation, are to be completed in “F4” and “F5”. After completing the filling of HCs in vehicles in “F6”, the carrier monitors the whole process of HCs vehicles on the road in “F7”. After unloading the HCs at the desired destination in “F8”, the HCs vehicle needs to be cleaned (“F9”), completing the transport process of HCs.

#### 5.1.2. Constructing the Function Network of HCRTS

**Figure 5:**
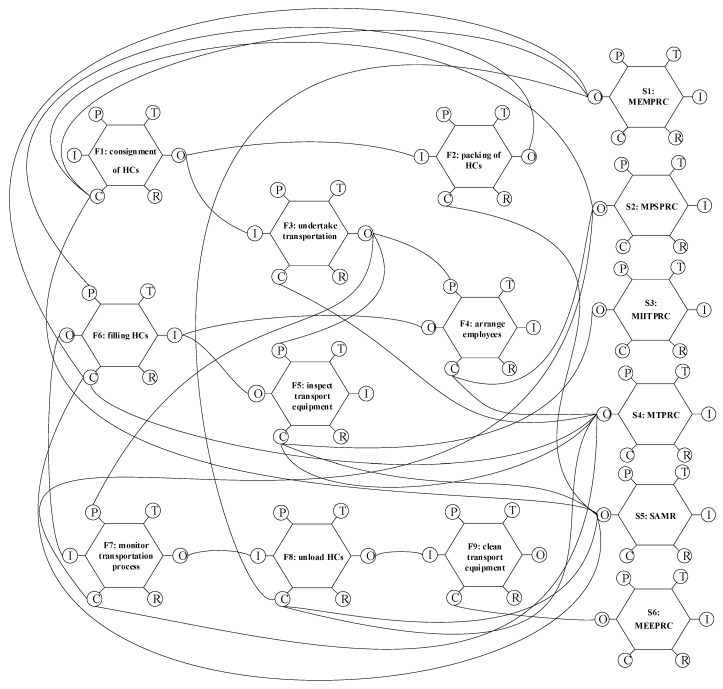
Function network of HCRTS by FRAM.

According to PSLPRCM, RSMHC, and SMMRTDG, the characteristic parameters of each function in the normal operation of the HCRTS are identified and sorted out, as shown in Appendix A Table A1. Then the identified functions are linked together graphically using the six aspects of the functions by FRAM, as shown in Figure 5. It is to be noted that, to explore and compare the regulatory effectiveness of regulatory bodies involved in different functions of HCRTS, the regulatory departments including MTMPRC, MPSPRC, MIITPRC, MTPRC, SAMR, MEEPRC are also added in the network, and linked with corresponding functions. As a result, there are a total of 29 function links in the function network, thus constituting the normal operation conditions of the HCRTS. From the perspective of functional resonance, an accident (or abnormal change of function) may occur because of the variabilities of its own function, or it may be the result of the coupling of upstream and downstream functions, that is, the variability of the upstream function leads to the variabilities of downstream function execution conditions (including input, output, time, control, preconditions, and resources), resulting in functional resonance and the failure of HCRTS. It can be seen that if the HCRTS is impacted, interfered with, or greatly varied in the normal operation process, the function links are affected, and then the whole HCRTS is in an unsafe state. Hence, to identify the failure function links, the risk factors representing failure function links exposed in accidents must be first recognized.

#### 5.1.3. Identifying the Failure Function Links in HCRTS

Firstly, to effectively identify the risk factors in the whole chain of HCRTS, an accident cause analysis was conducted by reviewing 94 HCs road transportation accidents data, sorting through preliminary risk factors. Secondly, several safety meetings included accident investigators, and certain people associated with the accidents were organized for correctness and rationality analysis of these preliminary risk factors. After several revisions and supplements in the process, a total of 32 risk factors were identified. Based on this, 12 failure function links in HCRTS were recognized by the categorization of these identified risk factors. It is to be noted that, since there was no accident involved in functions “F8”and “F9” in the collected data, the accident cause analysis was not able to finalise the risk factors. Table 2 presents the identified risk factors and their corresponding failure links in HCRTS.

### 5.2. Aggreating the Experts’ Judgements

After the risk factors that were categorized into failure function links were identified, this step sought to determine the weight of the likelihood and consequence of these risk factors. Firstly, a heterogeneous group of five experts, including an accident investigator, a HCs company operation manager, a senior engineer experienced in HCs transport equipment, a transportation safety senior scholar, and a HCs specialist, was employed to express their viewpoints on the consequence and likelihood of the risk factors based on linguistic terms. With rich experience in the safety of HCs road transportation, the weight of each expert is considered equal in this study. All the expert viewpoints on the risk factors in different function links are presented in Table 3.

After obtaining the judgements of marine experts, the weight of the likelihood and consequence of each risk factor was calculated by Equations (4) and (5), and the RI of each risk factor was procured by Equation (1). They are presented in Table 4.

### 5.3. Mapping the Risk Matrix

**Figure 6:**
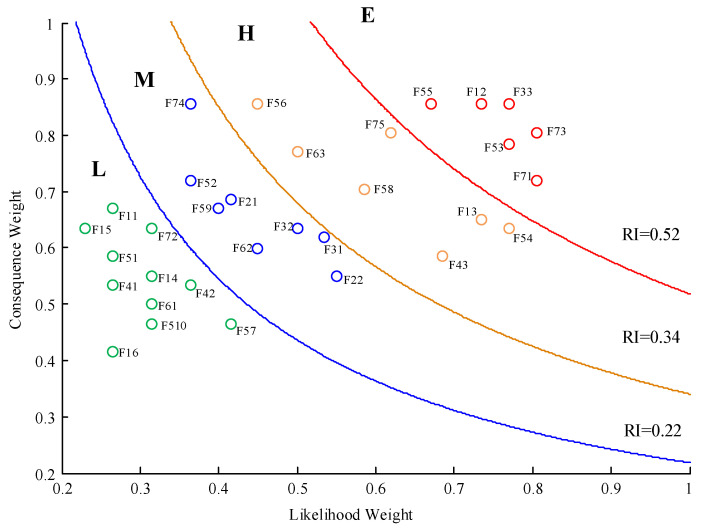
Two-dimensional risk matrix for risk factors in failure links.

According to the results provided in Table 4, a two-dimensional risk assessment matrix was constructed to assess the risk factors’ classes, as shown in Figure 6, in which consequence weight is depicted on the *x*-axis and likelihood weight on the *y*-axis. The matrix was divided into four groups that corresponded to four risk classes by three different isopleth index line of RI. Firstly, the yellow line with mean RI = 0.34 was found by averaging the total of 32 risk factors’ RIs, then the RIs of all risk factors was classified into two groups. One group with RI ≥ 0.34 contains 13 risk factors, by which the red line with mean RI = 0.52 was found by averaging these 13 risk factors’ RIs. Similarly, the blue line with mean RI = 0.22 was obtained by averaging the RI of risk factors in the other group with RI < 0.22. Therefore, all the identified risk factors were classified into four classes, namely, E (extreme risk), H (high risk), M (medium risk), and L (low risk). Specifically, there were six risk factors containing F33, F73, F12, F53, F71, and F55 classified as E; seven risk factors including F75, F54, F13, F58, F43, F63, and F56 belonged to H, eight risk factors consisting of F31, F32, F74, F22, F21, F62, F59, and F52 grouped into M; and the remaining risk factors belonged to E.

### 5.4. Results and Discussion

Figure 6 clearly illustrates the weight of likelihood and consequence, and the distribution of risk classification for the risk factors in the failure function links. According to the results in the matrix, the risk factors F73 and F71 have the highest likelihood weight, followed by F33, F53, and F54; the risk factors F33, F12, F55, F56, and F74 have the highest consequence weight, followed by F73, F75, and F53. This indicates that function links S2(O)-F7(C) and F3(O)-F7(P) are most likely to fail, while the failures of function links S4(O)-F3(C), F1(O)-F3(C), S3(O)-F5(C) and S2(O)-F7(C) follow. From the perspective of RI, six risk factors representing different failure function links are classified as extreme risk, and should be paid more attention to by HCs road companies, regulatory authorities, relevant practitioners, and related stakeholders. Risk factor F33 (affiliated operation and management) had the highest RI, followed by F73 (habitual illegal operation of the driver), and F12 (failure to check the qualification of the HCs vehicles). Since the RI of the risk factors can illustrate the strength of corresponding function links to a certain extent in HCRTS, the strength of function links could be ranked based on the assessment results. Hence, we can conclude that the function links S4(O)-F3(C), S2(O)-F7(C), F1(O)-F3(C), S3(O)-F5(C), and F3(O)-F7(P) are weaker in the HCRTS according to the risk factors in the first class. Based on these results, this paper also conducted post-interviews with these experts from the perspective of functional resonance to identify the deep-seated causes of these failure function links.

For function link F1(O)-F3(C), the highest contribution to its failure is the risk factor F12 (failure to check the qualification of the HCs vehicles). This is because the function F1 is intertwined with the upstream production and business activities of HCs, which directly determines the implementation conditions of the downstream transportation of HCs. The main reason for the failure of this link is that there is a blind spot in the supervision of MTPRC over the shippers (production and operation enterprises of HCs); moreover, there is a lack of effective communication between MEMPRC, SAMR, and MTPRC, which makes the risk factors in the process of production and operation of HCs an “import risk” to the HCRTS, and will cause the shock of functions in the downstream of HCRTS. As to the function links S4(O)-F3(C) and F3(O)-F7(P), the corresponding significant potential failures are manifested as the high-risk factors F33 (affiliated operation and management) and F71 (insufficient HCs vehicles dynamic monitoring), respectively. The main reason for the failure links is that the carrier evades—or is not well-supervised by—the MTPRC, which is reflected in affiliated operation and management by the HCs carrier, resulting in the responsibility of dynamic monitoring of HC vehicles on the road not being implemented well at the same time. For failure function link S3(O)-F5(C), the direct risk factors are F53 (illegal HCs vehicle modification) and F55 (illegal HCs vehicle modification). While the hidden cause is the lack of effective supervision of HCs vehicle enterprises by MIITPRC and SAMR, there is no effective communication and information transfer mechanism between MTPRC, MIITPRC, and SAMR, which enables a “connectivity risk” to stem from the information exchange barrier between different supervision departments to the HCRTS. For the function link S2(O)-F7(C), the risk factors mainly focus on the unsafe behavior of drivers, which are the direct causes of HCs road transportation accidents. From the view of upstream function output, the abnormal output of these functions, including employee arrangement, transportation equipment inspection, and HCs filling denotes the HCRTS operation “with disease”, which is the deep-seated cause for HCs road transportation accidents.

## 6. Conclusions

In the present study, a comprehensive mode that integrates FRAM, fuzzy sets, and risk matrix was proposed to evaluate the risk factors that represent failure function links in the whole chain of HCRTS. Among these, FRAM was used to construct a normal function network based on the function decomposition of HCRTS by HTA; the function links in HCRTS were then extracted using an operation network. To assess the risk factors, as well as corresponding failure function links, the risk factors were recognized first based on accident-cause analysis with the guidance of experts, and then fuzzy sets technology was adopted to obtain expert opinions on the weight of the likelihood and consequence of the identified risk factors. After aggregating the expert judgements, a two-dimensional risk assessment matrix was mapped to assess the risk factors’ classes, by which the weaker function links in HCRTS were identified. We concluded that F33, F73, F12, F53, F71, and F55 are the risk factors with higher RI, and the function links S4(O)-F3(C), S2(O)-F7(C), F1(O)-F3(C), S3(O)-F5(C) and F3(O)-F7(P) are weaker compared to other function links in HCRTS.

Although the proposed methodology integrating multiple technologies is an innovative attempt in assessing risk factors and function links, there are some limitations due to not making full use of the characteristics of FRAM technology. In future, potential work will be devoted to focusing on the quantitative assessment of function variability with FRAM, based on the accumulated data from a normal operation of HCRTS.

## Figures and Tables

**Table 1:** Linguistic expressions and corresponding TrFN.

Linguistic Expressions	TrFN
Very low (VL)	(0, 0, 0.1, 0.2)
Low (L)	(0.1, 0.25, 0.25, 0.4)
Medium (M)	(0.3, 0.5, 0.5, 0.7)
High (H)	(0.6, 0.75, 0.75, 0.9)
Very high (VH)	(0.8, 0.9, 1, 1)

**Table 2:** Risk factors and their corresponding failure links in HCRTS.

Function	Risk Factors	Failure Links
F1	F11—The shipper acquiesces to the transportation of non-conforming HCs vehicles	F1(O)-F3(C)
	F12—Failure to check the qualification of the HCs vehicles	
	F13—Failure to check the qualification of the drivers and escorts	
	F14—No production license for HCs	S5(O)-F1(C)
	F15—Beyond the scope of business of HCs enterprises	
	F16—Expired business qualification of HCs enterprises	
F2	F21—No MSDS (material safety data sheet) with HCs	S5(O)-F2(C)
	F22—HCs packaging not in conformity	
F3	F31—No qualification for HCs transportation	S4(O)-F3(C)
	F32—Illegal transportation of HCs beyond the scope	
	F33—Affiliated operation and management	
F4	F41—No qualifications of drivers and escorts	S4(O)-F4(C)
	F42—No escorts	F3(O)-F4(P)
	F43—Lack of safety education and training for employees	
F5	F51—No emergency shut-off valve installed	S3(O)-F5(C)
	F52—Illegal production and sale of HCs vehicles	
	F53—Illegal HCs vehicle modification	
	F54—Nonstandard inspection of HCs vehicles	
	F55—Illegal HCs tank installation	
	F56—Defects in HCs tank	
	F57—False test report of HCs tank provided by the third party	
	F58—No transport permit of the HCs vehicles	F3(O)-F5(P)
	F59—Not closed for the emergency shut-off valve	
	F510—Failed to timely repair the failed parts in HCs vehicles	
F6	F61—Filling HCs not in conformity with the notice	S4(O)-F6(C)
	F62—Irregular filling operation of HCs	
	F63—Overloading HCs	
F7	F71—Insufficient HCs vehicles dynamic monitoring	F3(O)-F7(P)
	F72—Not following the prescribed route	S4(O)-F7(C)
	F73—Habitual illegal operation of the driver	S2(O)-F7(C)
	F74—Overspeeding	
	F75—Fatigue driving	

**Table 3:** Linguistic judgements for the likelihood and consequence of risk factors.

Risk Factors	Likelihood					Consequence				
	E1	E2	E3	E4	E5	E1	E2	E3	E4	E5
F11	L	VL	L	M	L	VH	M	VH	H	L
F12	H	VH	H	H	M	H	VH	VH	H	VH
F13	M	H	H	VH	H	VH	VL	H	H	H
F14	L	VL	M	M	L	VH	VL	M	H	M
F15	VL	L	M	VL	L	H	M	VH	L	H
F16	L	VL	L	M	L	L	M	H	VL	M
F21	L	M	M	H	VL	H	VH	M	H	M
F22	M	H	L	H	M	L	H	L	H	H
F31	L	VH	H	L	M	VH	H	VH	L	L
F32	H	H	L	M	L	H	M	H	L	VH
F33	VH	H	VH	M	H	VH	H	VH	VH	H
F41	VL	L	M	L	L	H	L	VH	M	L
F42	L	VL	H	M	L	L	VH	L	H	M
F43	H	VH	M	H	M	VL	VH	H	VH	L
F51	VL	L	L	M	L	M	VH	L	M	H
F52	M	VL	L	H	L	VH	H	VH	M	M
F53	H	VH	VH	H	M	H	VH	H	H	H
F54	M	VH	H	H	VH	M	H	VH	M	M
F55	VH	H	VH	M	L	VH	H	VH	H	VH
F56	L	M	VH	M	VL	H	VH	VH	VH	H
F57	VL	M	L	H	M	VL	L	M	H	H
F58	M	H	VH	M	L	VH	VH	VH	L	M
F59	L	VL	VH	M	L	VH	L	VH	H	M
F510	VL	M	L	L	M	VL	H	L	M	H
F61	L	M	VL	M	L	VH	VL	H	L	M
F62	VH	VL	M	L	M	H	M	H	M	M
F63	M	L	M	M	H	VH	VH	H	M	H
F71	VH	H	VH	VH	M	VL	VH	VH	VH	H
F72	L	M	M	L	VL	VH	L	H	M	H
F73	VH	H	VH	VH	M	VH	M	VH	VH	H
F74	VL	H	L	M	L	H	VH	VH	VH	H
F75	M	VH	VH	L	M	VH	VH	H	M	VH

**Table 4:** RI of the risk factors.

Failure Function Links	Risk Factors	Likelihood Weight	Consequence Weight	RI
F1(O)-F3(C)	F11	0.27	0.67	0.18
	F12	0.74	0.86	0.63
	F13	0.74	0.65	0.48
S5(O)-F1(C)	F14	0.32	0.55	0.17
	F15	0.23	0.64	0.15
	F16	0.27	0.42	0.11
S5(O)-F2(C)	F21	0.42	0.69	0.28
	F22	0.55	0.55	0.30
S4(O)-F3(C)	F31	0.54	0.62	0.33
	F32	0.50	0.64	0.32
	F33	0.77	0.86	0.66
S4(O)-F4(C)	F41	0.27	0.54	0.14
F3(O)-F4(P)	F42	0.37	0.54	0.20
	F43	0.69	0.59	0.40
S3(O)-F5(C)	F51	0.27	0.59	0.16
	F52	0.37	0.72	0.26
	F53	0.77	0.79	0.60
	F54	0.77	0.64	0.49
	F55	0.67	0.86	0.57
	F56	0.45	0.86	0.38
	F57	0.42	0.47	0.19
F3(O)-F5(P)	F58	0.59	0.71	0.41
	F59	0.40	0.67	0.27
	F510	0.32	0.47	0.15
S4(O)-F6(C)	F61	0.32	0.50	0.16
	F62	0.45	0.60	0.27
	F63	0.50	0.77	0.39
F3(O)-F7(P)	F71	0.81	0.72	0.58
S4(O)-F7(C)	F72	0.32	0.64	0.20
S2(O)-F7(C)	F73	0.81	0.81	0.65
	F74	0.37	0.86	0.31
	F75	0.62	0.81	0.50

## Data Availability

The data that support the findings of this study are available from the corresponding author upon reasonable request.

## Appendix A. Function Parameters of HCRTS

**Table A1:** The six aspects of FRAM.

Function	Input	Output	Precondition	Resource	Control	Time
S1—consignment of HCs		Provide the HCs consignment list and road transport permit to carrier Check the qualification of carrier and HCs vehicle	Qualification of operating HCs	None	MEMPRC is responsible for safety supervision of the production, storage, use, and operation of HCs MPSPRC issues road transport pass for HCs SAMR issues the business license for the production, storage, operation, and transportation of HCs	
S2—packing of HCs	Consignment list	Finished packaging of HCs	Qualification of operating HCs	Packaging personnel/equipment	SAMR is responsible for supervising the product quality of packages and containers of HCs	
S3—undertake transportation	Consignment list	Finished waybill of HCs Safety education and training for employees Regular maintenance of HCs equipment Establishment of dynamic monitoring system for HCs	The carrier with HCs transport qualification	Knowledge of HCs transportation	MTPRC is responsible for examining and issuing HCs transport qualification	
S4—arrange employees	HCs waybill	Provide a driver and escort in each HCs vehicle	Drivers and escorts with qualifications Safety education and training for employees	Drivers and escort Safety education and training	MPSPRC issues driver’s license and supervises the allocation of escorts on HCs vehicle MTPRC issues driver qualification certificate of HCs for drivers and escorts	
S5—inspect transport equipment	HCs waybill	Completed the inspection of HCs equipment	HCs vehicle with road transport permit Regular maintenance of HCs equipment	Inspector	MIITPRC supervises and inspects HCs vehicle enterprises and vehicle quality SAMR supervises quality of HCs vehicle tanks MTPRC supervises the maintenance of HCs equipment	
S6—filling HCs	Finished inspection of HCs equipment	Completion of HCs filling	Finished packaging of HCs	Stevedore	MTPRC supervises the approval and record of HCs filling MEMPRAC and SAMR supervise the establishment of HCs filling procedures in companies	
S7—monitor transportation process	Completion of HCs filling	Real time transport route and driving behavior before arriving destination	Carrier implements dynamic monitoring of HCs vehicles	Monitoring platform and personnel on duty	MPSPRC is responsible for the management of traffic order of HCs vehicles MTPRC supervises the dynamic monitoring work of HCs in enterprises	
S8—unload HCs	Arrival of destination	Completion of HCs unloading	Establish HCs operation and record system.	Stevedore	MTPRC shall supervise the approval and record of unloading HCs MEMPRAC and SAMR supervise the establishment of HCs filling procedures in company	
S9—clean transport equipment	Completion of HCs unloading	Complete the cleaning of HCs transportation equipment	None	Cleaner	MEEPRC supervises the discharge of HCs wastewater	
